# Supplementary figures and images for: Effect of Feeding Cold-Pressed Sunflower Cake on Ruminal Fermentation, Lipid Metabolism and Bacterial Community in Dairy Cows
Source: Animals (Basel). 2019 Oct 1;9(10):755. doi: 10.3390/ani9100755 (PMC6826361; doi:10.3390/ani9100755)

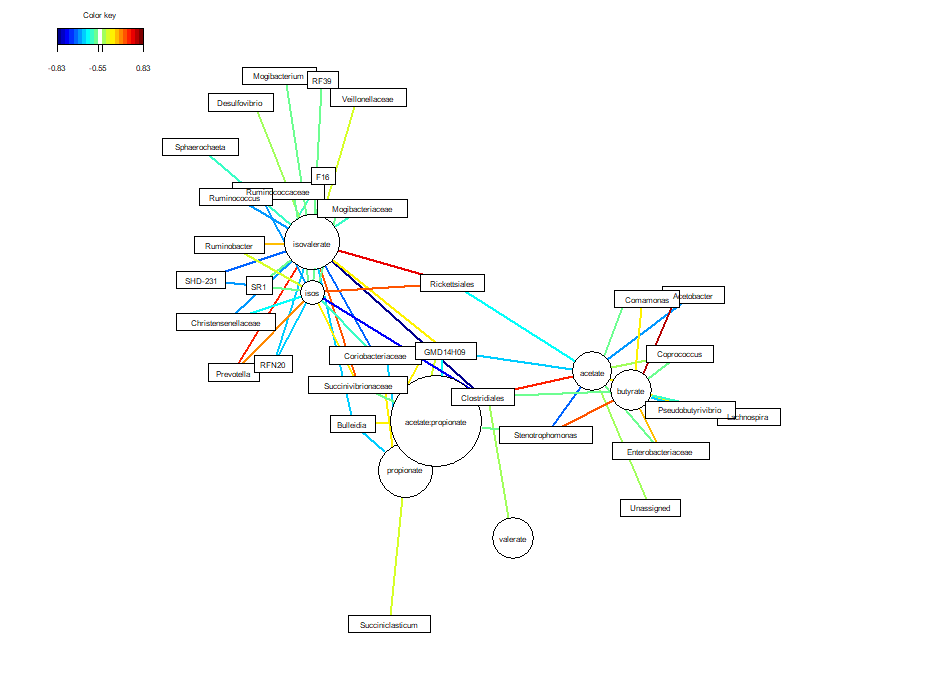

Supplement: Supplementary file 1 [file animals-09-00755-s001.zip › Supllementary FigureS2.png]

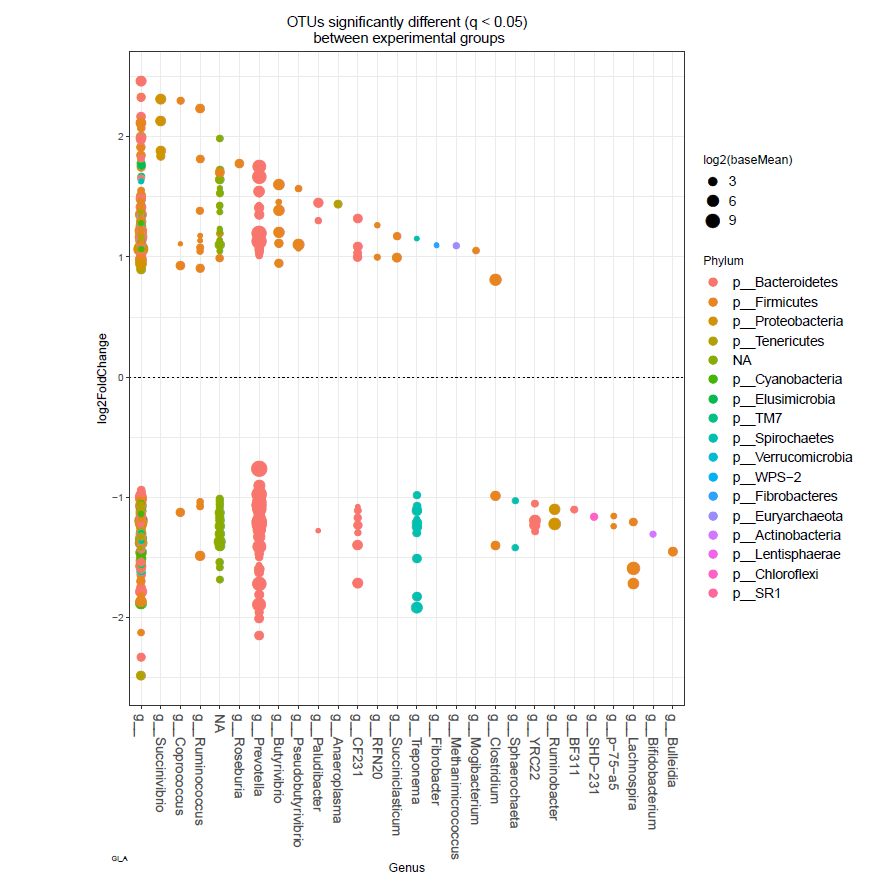

Supplement: Supplementary file 1 [file animals-09-00755-s001.zip › Supplementary FigureS1.png]
